# Supplementary material for: Do genetic ancestry tests increase racial essentialism? Findings from a randomized controlled trial
Source: PLoS One. 2020 Jan 29;15(1):e0227399. doi: 10.1371/journal.pone.0227399 (PMC6988910; doi:10.1371/journal.pone.0227399)
Supplement: S1 Appendix — (DOCX) [file pone.0227399.s001.docx]

**Supporting Information**

**Data and Methods**

Data for this study come from an original, unblinded, parallel arm randomized controlled trial testing the impact of genetic ancestry testing on genetic essentialist views of race. We hired NRG Research Group to recruit participants using random digit dialing across the continental U.S. to screen for eligibility. Individuals were eligible if they were born in the United States, self-identified as non-Hispanic White, were age 19 or older, neither they nor an immediate family member had taken a genetic ancestry test, and they were willing to take a genetic ancestry test. We stratified our sampling by gender, age, education, and region based on the national population of native-born non-Hispanic Whites aged 19 and older to improve the demographic diversity of our sample. NRG called landline and cellphone numbers and assessed 4,191 people for eligibility. Of those, 40.9% (N=1,716) were deemed eligible for the study. Following the establishment of eligibility, NRG told prospective participants in general terms what the study involved and asked for their email address if they were interested in being sent more information and detailed consent forms by the researchers; 90.3% of those eligible agreed to be contacted (N=1,550).

Because the study and its risks and benefits required long consent forms, the University of British Columbia Behavioral Research Ethics Board, which approved the study, requested that treatment and control groups receive tailored consent forms to minimize length and promote legibility. Random allocation into the control group (N=783) or the treatment group (N=767) therefore occurred when prospective participants provided their email address. Using a random number generator, we applied a randomly generated series of 0s (control) and 1s (treatment) to the individuals in the order in which they were recruited by NRG, using a 1:1 ratio, to determine which consent form an individual would receive. Because group assignment is not blind to participants, the allocation was not concealed from researchers.

We offered small financial incentives for completing each online survey: $15 (USD) to both groups, as a check or gift card, for completing the pre-test survey. Treatment respondents were offered a $5 Starbucks gift card for completing the First Reactions Survey. We offered a $15 incentive to the treatment group and a $20 incentive to the control group for completing the post-test survey. Approximately three weeks before the end of post-test survey data collection, we emailed participants who had not yet completed and offered an increased incentive of $25 for treatment and $30 for control respondents. This decision drew on findings that the views of people who agree to participate but later do not complete all study segments may differ from those of other respondents, and such efforts can address this issue [1,2].

We asked those assigned to the control group not to take genetic ancestry tests before the end of the study. We informed them that they would receive a discount coupon to purchase the same tests at half price after the study, providing an additional incentive to participate and a motivator to not purchase the tests while the study was in progress. At this point, 64.1% of those allocated to the control group (N=502) and 65.8% of those allocated to the treatment group (N=505) consented to participate and completed the online pre-test survey, between October 2014 and February 2015.

When those in the treatment group completed their pre-test survey, we mailed them a test kit, with instructions for them to send a DNA sample directly to the testing company. We used Family Tree DNA, one of the largest DTC genetic ancestry testing companies, so that the test reports would be comparable to what many test consumers receive. We also chose a company that does not provide estimates of genetic disease risk due to additional ethical concerns in providing such information. Admixture and mtDNA tests (Family Tree DNA’s Family Finder test and mtFull Sequence test, respectively) were conducted on the treatment respondents’ samples. Admixture tests attempt to infer someone’s geographical origins based on an analysis of their ancestry across their genome, by comparing nuclear DNA to a panel of single-nucleotide polymorphisms that are considered to be ‘ancestry informative markers’, although those markers are not uniquely held by the populations they are used to indicate. Mitochondrial, or mtDNA, tests trace a person’s direct matrilineal ancestry using the DNA in her or his mitochondria; when the respondent’s sample and reference DNA match at a number of markers, she or he is considered to share a distant maternal ancestor with the person who was the source of the reference sample [3]. We chose mtDNA tests because both female and male respondents could take them, unlike Y-DNA tests.

When both sets of tests were complete, we emailed the respondent a link to view their online test results, and reported their admixture breakdown in the email. Both the mtDNA and admixture test results included several webpages and links that respondents could view online. S1 Fig and S2 Fig show examples of test results. Although these figures are only part of what was available, we asked treatment respondents to view these specific figures. We also asked them to spend at least half an hour looking through their test results and then to take a short “First Reactions” survey. This survey helped ensure that they looked at their test results and provided data on their initial reactions. They completed this survey between January-July 2015.

Both the treatment and control groups were invited to take an online post-test survey 11 months after they completed their pre-test survey. These were completed between September 2015 and March 2016. Nine control respondents were excluded from analysis because they (N=3) or a relative (N=6) had taken a genetic ancestry test since the date of their pre-test survey and they were aware of the test results. The final analytical sample is comprised of 802 participants (control N=425; treatment N=377). The completion rate was 84.7% of control participants and 74.7% of treatment participants. Fig 1 provides a flow chart for the study design and response, following the CONSORT 2010 RCT reporting guidelines [4]. Descriptive statistics for the control and treatment groups are shown in S2 Table on the right (“Remaining Participants”).

After data collection, we sent all participants a debriefing email on study goals. This provided a sociological perspective that race is not purely determined by genetics and explained social scientists’ concerns that some people might interpret genetic ancestry tests as reifying race, as well as others’ suggestions that it could have the opposite effect, and our attempt to answer this question empirically. It also discussed some of the tests’ limitations, and the first author’s views on how the tests are frequently misinterpreted as well as ways that they can be useful.

**Analysis of Attrition**

While there is no absolute standard for acceptable levels of attrition, many agree that bias is primarily a concern if attrition surpasses 20% [5,6]. Ours does not surpass this level, but we analyze both overall and differential attrition between the treatment and control groups as a precaution, given their importance for longitudinal studies in general and RCTs in particular.

To analyze overall attrition, we use logistic regression analysis to estimate differences between those who left the study and those who remained, presented as odds ratios for remaining [7]. In S1 Table, Model 1 includes our control variables and the 4-category genetic knowledge scale. It shows that people with a greater knowledge of genetics have greater odds of remaining in the study, likely out of interest in its topic. Notably, however, when we run separate models (not shown) with dummy variables, neither people with high genetic knowledge nor no genetic knowledge have significantly greater (or lower) odds of remaining in the study. Rather the effect appears in the middle ranges; those with low genetic knowledge have significantly greater odds of dropping out, and those with medium genetic knowledge have significantly greater odds of remaining. Model 1 also shows that people age 55 and above have greater odds of completing the study (relative to people age 19-34). Model 2 adds the pre-test Genetic Essentialism Scale for Race; respondents’ initial beliefs in genetic essentialism do not significantly influence their odds of remaining in the study. Model 3 adds assignment to the treatment group and shows that treatment respondents have significantly lower odds of remaining in the study, even with other factors held constant. We expected greater attrition in the treatment group because it involves the extra steps of sending a DNA sample in to the company, viewing test results online, and taking the additional First Reactions survey.

Greater attrition in the treatment group would be a concern primarily if there were differential attrition between the control and treatment group with regard to the characteristics of who remains in the study, however our analysis shows that there is not. In S2 Table we report the baseline characteristics of all initial participants, those who are lost to follow-up, and those who remain in the study to completion [5]. We analyzed differences between the control and treatment groups in each of these three conditions using independent sample two-tailed t-tests. Among all initial participants, there are no significant differences between control and treatment groups on any of our measures, as would be expected with successful randomization. After agreeing to participate, more people who were age 55 and above and who had greater interactions with non-Whites dropped out of the study in the control group than in the treatment group. Yet these differences in the participants lost to follow up do not create significant imbalances among the remaining participants. None of our measures show statistically significant differences between control and treatment groups among those who complete the study and are the focus of our analysis. Attrition has not affected the reliability of our comparisons between the treatment and control groups.

**Comparison of Sample to US-Born Non-Hispanic White Population**

Our target population is not all US-born non-Hispanic Whites, but those who were willing to take genetic ancestry tests and had not previously received personalized genetic ancestry information. Yet to assess how they differ, we compared the demographic characteristics of our sample to that of a representative sample of all US-born non-Hispanic Whites aged 19 and older in the American Community Survey (ACS). The ACS is an annual nationwide survey conducted by the U.S. Census Bureau that measures demographic, social, and economic attributes of the U.S. population. The ACS sample is a random sample of 3.5 million U.S. households [8]. For this analysis, we use the 1-year estimate from 2015, which was collected in approximately the same year that our project was conducted.

Frequencies comparing the ACS sample with our sample are displayed in S3 Table. Compared to the American Community Survey sample, our study sample has a greater proportion of female participants (63.3% vs. 51.2% of the ACS sample) and a higher overall level of educational attainment (32.4% of our sample has more than college, compared to 11.5% of the ACS sample). To match the ACS sample, our sample would need fewer participants with advanced graduate degrees and a greater proportion of participants with a high school education or less. Our study sample also has a smaller proportion of participants under 34 years of age, however both samples contain a significant proportion of participants aged 55 or older. With regard to region, the current study sample demographics are similar to those of the ACS sample. These differences likely reflect what types of people are willing to take genetic ancestry tests, although they may also indicate demographic tendencies in willingness to participate in a research study.

We also compared the genetic knowledge of our sample to that of a representative sample of non-Hispanic White adults in the Survey on Genomics Knowledge, Attitudes and Policy Views (GKAP) (bottom of S3 Table). More of our respondents answered the questions that contribute to our genetic knowledge scale correctly than did respondents in the GKAP survey. On the first question, “Based on what you know, would you say that DNA can be found in every cell in the human body or only in specific organs and cells in the human body?” 92.3% of our sample answered correctly compared with 76.7% of the GKAP sample. On the second question, “Based on what you know, would you say that more than half, about half, or less than half of a human being’s genes are identical to those of a mouse?” 34.5% of our sample answered correctly compared with 19.5% of the GKAP sample. We suspect that people who are willing to take genetic ancestry tests have more knowledge of basic genetics than the population in general.

**Dependent Variable: Genetic Essentialism Scale for Race (GESR)**

We developed the GESR using exploratory and confirmatory factor analysis. The creation and validation of the scale is described in detail elsewhere [9].We extracted GESR as a second-order construct using the ‘predict’ command after we ran a structural equation model (command: ‘sem’) in Stata 14.2. We generated the scale on the sample who completed both the pre-test and post-test surveys to ensure consistency across samples used for the pre-test and post-test GESR. The items used in the model are listed in S4 Table. The command is as follows:

sem (CoreDeterminism-> Athlete Smart Pure) ///

(CategoryDeterminism -> Popdivide GeneticsTells Classify) ///

(Polygenism -> RaceInvent AllAfrican Allshare) ///

(GESR-> CoreDeterminism CategoryDeterminism Polygenism), ///

var (e.GeneticEssentialism@1) ///

latent (CoreDeterminism CategoryDeterminism Polygenism GESR) ///

standardized nocapslatent

We also ran the same model with ‘gsem’ command, which is meant for generalized structural equation models; the results were the same and are available upon request.

We used mixed-models in our experimental analysis, which requires a long-shaped dataset. After we reshaped the dataset from wide to long, we rescaled the GESR to range from 0 to 1.

**Genetic Knowledge Variables**

The survey had three questions to measure factual genetic knowledge, taken from the Survey on Genomics Knowledge, Attitudes and Policy Views [10]. One of these questions had close resemblance with the items that made up the GESR: (3) Based on what you know, would you say that more than half, about half, or less than half of a white person’s genes are identical to those of a black person? (67.8% correctly answered “more than half”). Because this question can be a measure of belief in genetic essentialism, specifically the sub-dimension we have elsewhere called “category determinism” [11], we omitted it from our genetic knowledge scale. However, models that included this item showed similar results. The distribution of the responses to the two questions we used is shown in S5 Table.

The correct answer for both of the questions shown in S5 Table is the first option. Because the questions vary in difficulty, we developed weights to factor in the parameter of difficulty. For instance, while 91.86% answered the first question correctly, only 32.87% answered the second question correctly. We used the percentages of people who answered the question correctly as a proxy for their difficulty. We assigned ½ point to answers that were closer to the correct answer for the second question, to give partial credit to those individuals. “Don’t Know” responses were treated as equivalent to “No Knowledge” and assigned the weight of “0.”

We first calculated the total number of points given for these two questions by multiplying the percentage of people in each response category and the point value assigned to each response category. The weights applied are then as follows:

Q1: 1*91.86+ 0*0.89 +0*7.25=91.86 total points

Q2: 1*32.87 +0.5*10.23+0*16.98+0*39.92=37.99 total points

We generated binary variables for each category of each question, and multiplied each of these binary variables by the inverse of the total points calculated for each question so that each response category was further weighted by how accurate the response was.

Next, we added all the binary variables representing response categories for the two questions together. This produced raw scores in 6 categories. We considered what level of measurement would be best for the genetic knowledge variable. Given the scale nature of our composite index, a continuous variable was the first option we entertained. Yet, given that there are only four effective categories out of the six in the full range (as two of the categories have very few cases in them), we did not think it would be a viable option. When categories are few, it is not recommended to treat a categorical variable as continuous [12]. Furthermore, the variable is not normally distributed, and we expected that it would not be reasonable to treat the variable as continuous and assume that the one-unit effect would be the same across different points of the scale. We next considered an ordinal variable, which we created by collapsing the 6 raw categories to 4 categories because 2 of them had very few cases. We used this variable in our primary analysis. As a robustness check, we further collapsed these 4 categories to 2, creating an additional dichotomous version of this variable [13]. The raw scores, the 4-category ordinal genetic knowledge measure, and the dichotomous genetic knowledge measure are shown in S6 Table.

**Coding of Known Ethnicity and Admixture Test Results**

In the non-experimental analysis focusing only on the treatment respondents, we considered the role of participants’ knowledge of their own ancestry and their genetic ancestry test results in shaping genetic essentialism. First, we numerically coded each ethnic identity entry listed by participants in the pre-test survey according to the categories provided in the U.S. Census Bureau’s American Community Survey (ACS) ancestry code list [14]. In the pre-test survey, participants were also asked to list all the ethnic ancestries of each of their biological parents. Following the same procedures, we coded the ancestries listed for participants’ parents using the ACS code list. To account for instances where a participant had knowledge of an ethnic ancestry but did not identify with it and list it among their own ethnic identities, we created a new variable that combined both their own ethnic identities and their parents’ ethnic ancestries to capture the ethnic ancestries of which they had knowledge.

The admixture test results reported ancestry in the following categories: British Isles, Western and Central Europe, Eastern Europe, Southern Europe, Scandinavia, Finland and Northern Siberia, Asia Minor, Middle East, Eastern Middle East, Ashkenazi Diaspora, Africa, North Africa, West Africa, East Central Africa, South Central Africa, East Asia, Central Asia, Central South Asia, Northeast Asia, and Native American. The tests also occasionally included aggregated categories when the results could not distinguish between them (e.g. British Isles, Western and Central Europe; Scandinavia, Western and Central Europe). For all the ancestry categories, we created binary variables where a value of 1 indicated that the respondent’s admixture test reported any ancestry in that category.

We aggregated participants’ ACS-coded known ethnic ancestries to correspond to the broad regional ancestry categories reported by Family Tree DNA and compared their pre-test knowledge of their ancestries (their ethnic identities and their parents’ ancestries) to their genetic ancestry admixture test results. We created one set of variables to indicate that a particular ancestry was listed by the participant as known in the pre-test survey and was subsequently indicated in the admixture test results – in other words, an indicator of whether a particular known ancestry was “confirmed” by the test. A second set of variables denotes participants whose admixture tests reported a specific ancestry that did not correspond to any known ancestry they listed in the pre-test survey – in other words, they “discovered” a new ancestry from their test that was not previously known. By incorporating these two sets of variables into the analysis, we account for both previous knowledge of personal ancestry and the receipt of a “confirmatory” or “revelatory” genetic ancestry test in shaping essentialist views. Descriptive statistics for these variables, grouped into European and non-European ancestries, is shown in S7 Table. We included Ashkenazi Diaspora as a European ancestry for this division; additional analyses (not shown) separating it from the European category did not change the results. Our coding is available upon request.

**Control Variables**

Our models control for living in the South, interaction with non-Whites and political party preference, as well as gender, age, and education. We control for living in the South as we expect the legacy of slavery and Jim Crow there may have led to higher genetic essentialist views of race [9,15]. For “South” we use the same definition as the U.S. Census Bureau statistical regions. Specifically, the South census region includes Alabama, Arkansas, Delaware, the District of Columbia, Florida, Georgia, Kentucky, Louisiana, Maryland, Mississippi, North Carolina, Oklahoma, South Carolina, Tennessee, Texas, Virginia, and West Virginia [8]. Interaction with non-Whites influences Whites’ racial attitudes and may also affect views of genetic essentialism [9,16,17]. This variable is a composite measure averaging the responses from respondents’ reported frequency of having a conversation with someone who is Black, Asian, Latino, Native American or Middle Eastern, respectively, over the past 6 months; the 7-point response scale ranges from “not at all” to “every day.” For political party preference, which is associated with essentialism [9,18], we asked how favorable or warm respondents feel toward the Republican Party and the Democratic Party, respectively (from 0=not at all to 10=very favorable or warm). Because they are correlated (r=-.55), we created a difference scale of feelings toward Republicans minus feelings toward Democrats, for a measure indicating relative preference for the Republican Party. See S2 Table on the right (“Remaining Participants”) for summary statistics.

**Statistical Analysis**

In our experimental design, there were two fixed effects (treatment and time) and two sources of random variation (between and within individuals). To account for the correlations between the observations made on the same individual and possible heterogeneous variances among observations on the same individual over time, we used a linear mixed-effects model (LMM) to examine the causal effect of taking the ancestry tests [19]. Even though the control and treatment groups were very similar in terms of distribution of variables in the pre-test survey, for any slight differences that existed, we were able to rely on LMM because it allows for pre-treatment mean differences between the groups.

**Model:** For the analysis of the treatment effect, we fitted the following linear mixed effects model. Formally the model is:

$$Y_{ij=} \beta_{0}+ \beta_{1}G_{i} + \beta_{2}T_{i} +\beta_{3}G_{i}T_{i} + \gamma X_{i}+u_{i} + \varepsilon_{ij}$$

where

Y_ij_ denotes the response variable scores for subject i at time j;

β_0_ is the mean response;

G_i_ refers to Group—Control (0), Treatment (1);

T_j_ refers to pre-test (0) or post-test (1);

X_i_ is the vector of control variables all measured in time 0;

u_i_ is the random intercept at the individual level;

ε_ij_ is the error term for subject i at time j_._

E(ε_ij_)=0_,_ Var(ε_ij_)=σ^2^; E(u_j_)=0_,_ Var(u_j_)=τ^2^; Cov(ε_ij,_u_j_)=0.

S8 Table shows the mixed model where we ran interactions of genetic knowledge and assignment to the treatment group. Because it is hard to read interaction effects from the regression output in three-way interactions, we ran pairwise comparisons to calculate the marginal change in each genetic knowledge group between pre- and post-treatment (S9 Table). We also ran Ordinary Least Squares (OLS) regression models on the post-test genetic essentialism scale while controlling for the pre-test genetic essentialism scale (S10 Table) to verify the results from the LMM model.

For our additional analysis using a dichotomous genetic knowledge measure as a robustness check, we present the pairwise contrasts of genetic essentialism scores between pre-test and post-test for groups with lower and higher genetic knowledge levels in S11 Table. For comparison to other figures, these results are graphed in S3 Fig.

For the analysis that looks at the treatment group only, we used OLS models with “confirmation of a known ancestry” and “discovery of a new ancestry” as the two main independent variables in separate models, controlling for the same list of variables: genetic knowledge, region (South), interaction with non-Whites, political party preference, and basic demographics (age, gender, education) (see S7 Table for descriptive statistics). These models similarly regress the post-test genetic essentialism scale while controlling for the pre-test genetic essentialism scale. We ran separate models for the effect of “confirming” a European (S12 Table) and a non-European ancestry (S13 Table); we also ran combined models for the effect of “confirming any ancestry” (not shown). Similarly, we distinguished between “discovering” a European and a non-European ancestry (S14 Table). Beyond the baseline models, we ran interaction models examining whether the effect of “confirming a known ancestry” or “discovering a new ancestry” changes with varying genetic knowledge levels. Full model results are available upon request.

In all the models, both experimental and non-experimental, we controlled for genetic knowledge, region (South), interaction with non-Whites, political party preference, and basic demographics (age, gender, education).

**References**

1. Booker CL, Harding S, Benzeval M. A systematic review of the effect of retention methods in population-based cohort studies. BMC Public Health. 2011;11: 249. doi:10.1186/1471-2458-11-249

2. Brueton VC, Tierney JF, Stenning S, Meredith S, Harding S, Nazareth I, et al. Strategies to improve retention in randomised trials: a Cochrane systematic review and meta-analysis. BMJ Open. 2014;4: e003821. doi:10.1136/bmjopen-2013-003821

3. Nelson A. Bio Science: Genetic Genealogy Testing and the Pursuit of African Ancestry. Soc Stud Sci. 2008;38: 759–783. doi:10.1177/0306312708091929

4. Schulz KF, Altman DG, Moher D. CONSORT 2010 statement: Updated guidelines for reporting parallel group randomised trials. Int J Surg. 2011;9: 672–677.

5. Dumville JC, Torgerson DJ, Hewitt CE. Reporting attrition in randomised controlled trials. BMJ. 2006;332: 969–971.

6. Marcellus L. Are We Missing Anything? Pursuing Research on Attrition. CJNR Can J Nurs Res. 2004;36: 82–98.

7. Miller RB, Hollist CS. Attrition Bias. In: Salkind NJ, editor. Encyclopedia of Measurement and Statistics. Thousand Oaks, CA: SAGE Reference; 2007. pp. 57–60.

8. US Census Bureau. American Community Survey (ACS). 2019 [cited 13 Nov 2019]. Available: https://www.census.gov/programs-surveys/acs

9. Yaylaci S, Roth WD, Jaffe K. (July 2019) Measuring Racial Essentialism in the Genomic Era: The Genetic Essentialism Scale for Race (GESR). Curr Psychol. doi:10.1007/s12144-019-00311-z

10. Hochschild J, Sen M. Genetic Determinism, Technology Optimism, and Race Views of the American Public. Ann Am Acad Pol Soc Sci. 2015;661: 160–180. doi:10.1177/0002716215587875

11. Yaylacı Ş, Roth WD, Jaffe K. Measuring racial essentialism in the genomic era: The genetic essentialism scale for race (GESR). Curr Psychol. 2019 [cited 20 Aug 2019]. doi:10.1007/s12144-019-00311-z

12. Liddell TM, Kruschke JK. Analyzing ordinal data with metric models: What could possibly go wrong? J Exp Soc Psychol. 2018;79: 328–348. doi:10.1016/j.jesp.2018.08.009

13. MacCallum RC, Zhang S, Preacher KJ, Rucker DD. On the practice of dichotomization of quantitative variables. Psychol Methods. 2002;7: 19–40. doi:10.1037/1082-989X.7.1.19

14. US Census Bureau. American Community Survey and Puerto Rico Community Survey 2017 Code List. Washington, D.C.; 2017. Available: https://www.census.gov/programs-surveys/acs/technical-documentation/code-lists.html

15. Davis FJ. Who Is Black? One Nation’s Definition. University Park, PA: Pennsylvania State University Press; 1991.

16. Pettigrew TF, Tropp LR, Wagner U, Christ O. Recent advances in intergroup contact theory. Int J Intercult Relat. 2011;35: 271–280. doi:10.1016/j.ijintrel.2011.03.001

17. Tawa J. Belief in Race as Biological: Early Life Influences, Intergroup Outcomes, and the Process of “Unlearning.” Race Soc Probl N Y. 2016;8: 244–255. doi:http://dx.doi.org/10.1007/s12552-016-9176-7

18. Morin-Chassé A, Suhay E, Jayaratne TE. Discord Over DNA: Ideological Responses to Scientific Communication about Genes and Race. J Race Ethn Polit. 2017;2: 260–299. doi:10.1017/rep.2017.17

19. Moskowitz DS, Hershberger SL, editors. Modeling Intraindividual Variability with Repeated Measures Data : Methods and Applications. New York: Routledge; 2002. doi:10.4324/9781410604477
