# Supplementary figures and images for: Do genetic ancestry tests increase racial essentialism? Findings from a randomized controlled trial
Source: PLoS One. 2020 Jan 29;15(1):e0227399. doi: 10.1371/journal.pone.0227399 (PMC6988910; doi:10.1371/journal.pone.0227399)

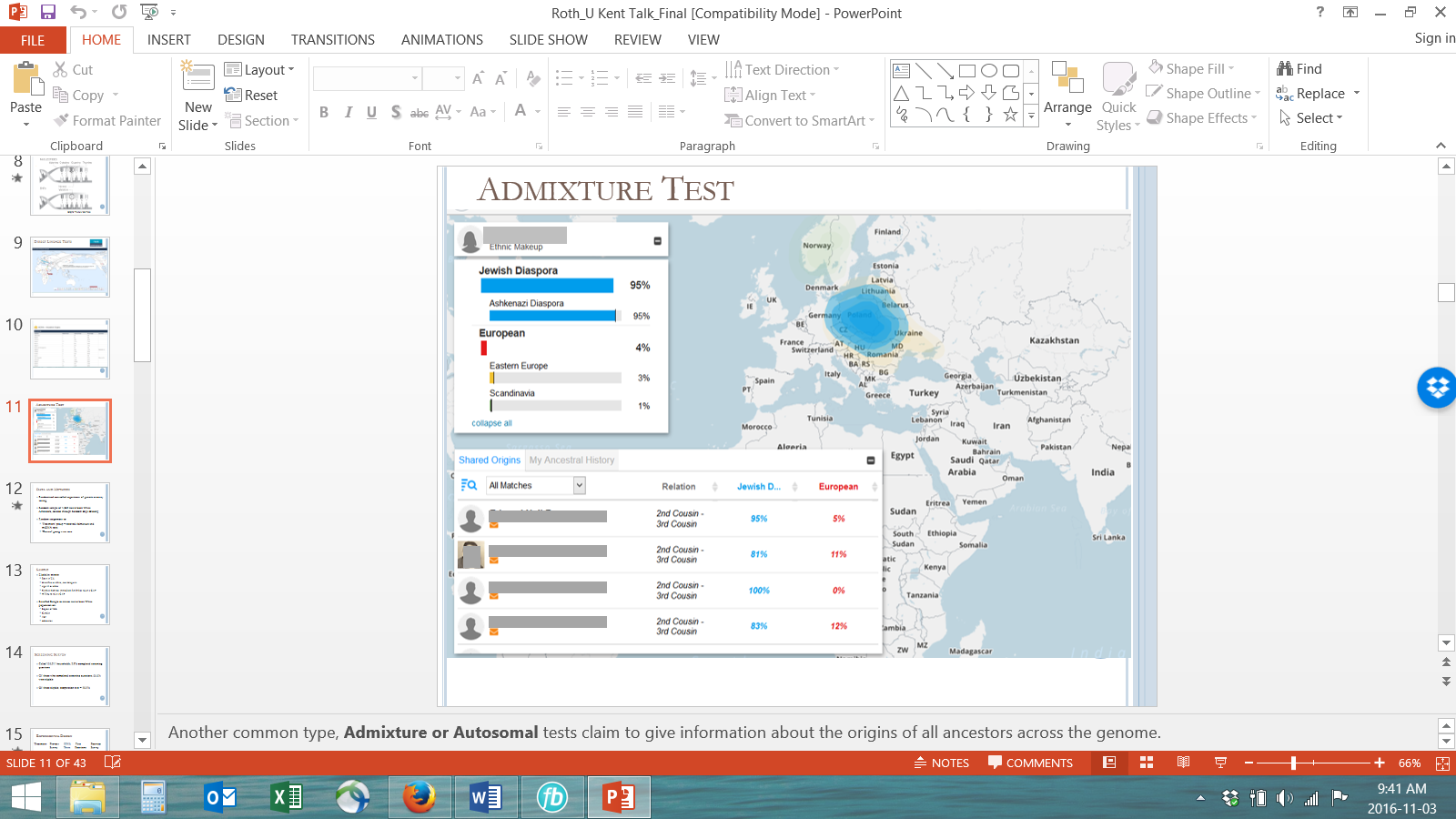

Supplement: S1 Fig — Figure Credit: Family tree DNA. (DOCX) [file pone.0227399.s002.docx]

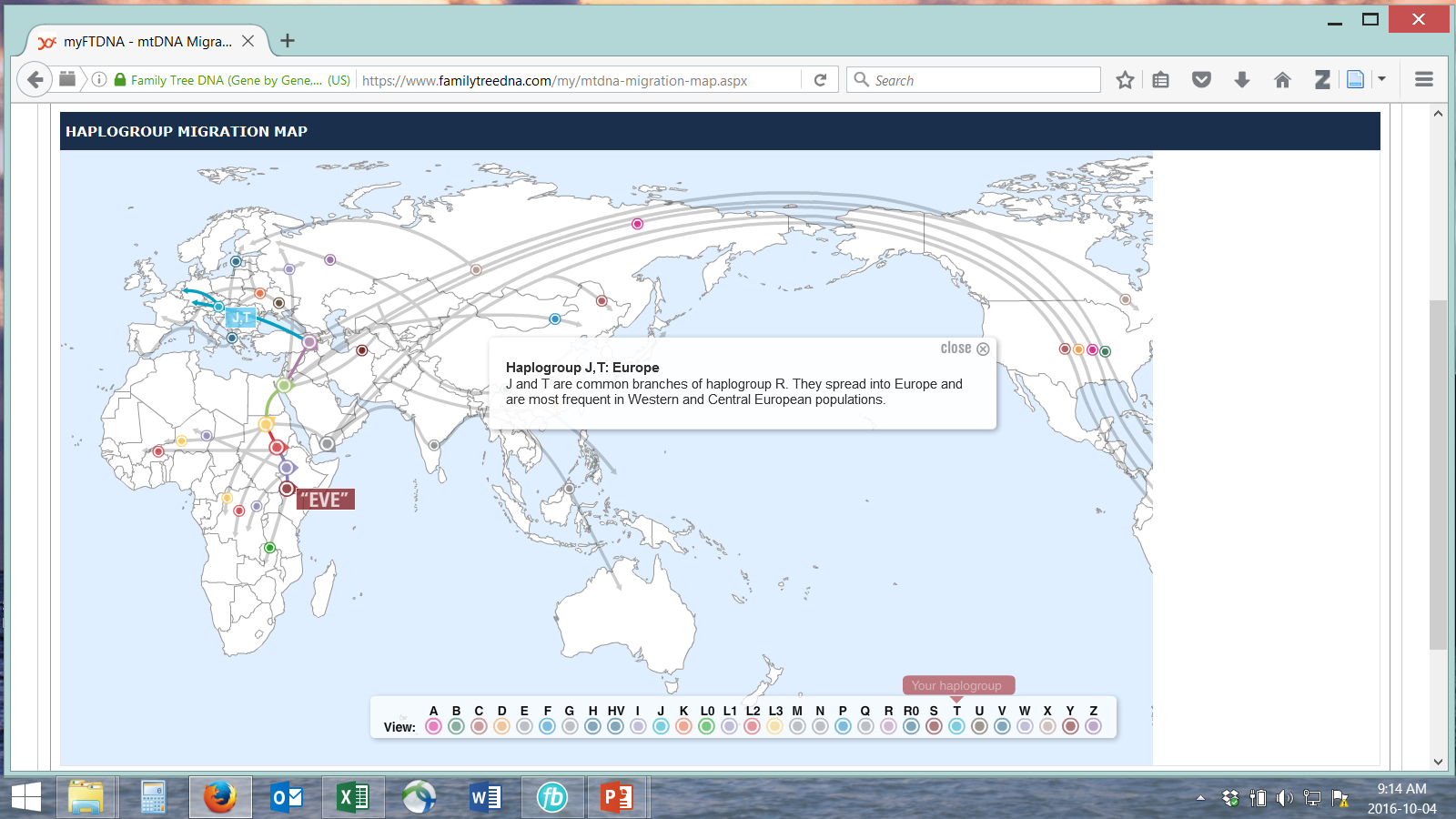

Supplement: S2 Fig — Figure Credit: Family tree DNA. (DOCX) [file pone.0227399.s003.docx]

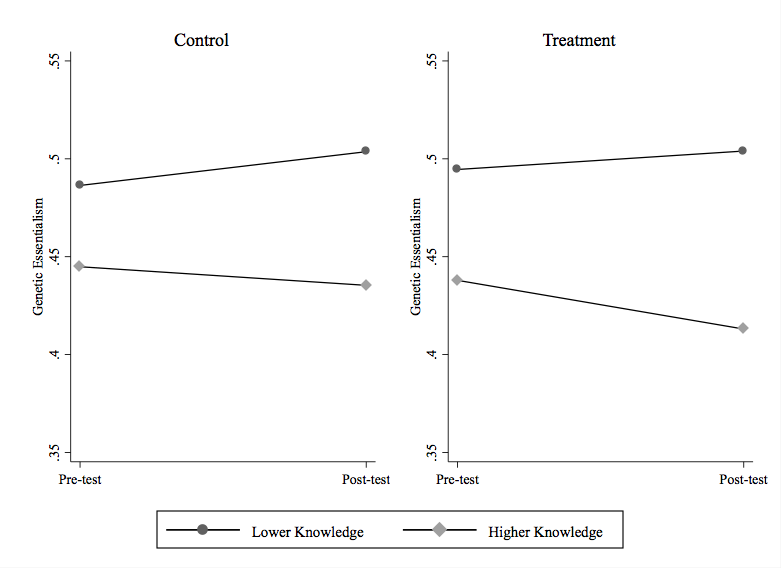

Supplement: S3 Fig — This graph plots the interaction of genetic knowledge measured as a dichotomous variable and the study arm allocation group variables. It shows the predicted average change in the pre-test and post-test genetic essentialism scores of those with lower and higher genetic knowledge within the control and treatment groups separately. We used Stata’s “margins” command to produce the graph. (TIF) [file pone.0227399.s004.tif]
