# Supplementary material for: Do genetic ancestry tests increase racial essentialism? Findings from a randomized controlled trial
Source: PLoS One. 2020 Jan 29;15(1):e0227399. doi: 10.1371/journal.pone.0227399 (PMC6988910; doi:10.1371/journal.pone.0227399)
Supplement: S1 Table — (DOCX) [file pone.0227399.s005.docx]

|  | Model 1 | | Model 2 | | Model 3 | |
| --- | --- | --- | --- | --- | --- | --- |
|  | Odds ratios | (S.E.) | Odds ratios | (S.E.) | Odds ratios | (S.E.) |
| Genetic Knowledge  (Omitted = High knowledge) |  |  |  |  |  |  |
| No knowledge | 0.735 | (0.347) | 0.791 | (0.351) | 0.766 | (0.353) |
| Low knowledge | 0.642* | (0.194) | 0.683 | (0.198) | 0.680 | (0.199) |
| Medium knowledge | 1.003 | (0.304) | 1.055 | (0.306) | 1.049 | (0.308) |
| Pre-test Genetic Essentialism |  |  | 0.434 | (0.544) | 0.431 | (0.551) |
| Treatment Group |  |  |  |  | 0.560*** | (0.164) |
|  |  | |  | |  | |
| Male (Omitted = Female) | 1.065 | (0.170) | 1.059 | (0.170) | 1.058 | (0.171) |
| Age (Omitted = 19-34) |  |  |  |  |  |  |
| 35-54 | 1.430 | (0.251) | 1.440 | (0.252) | 1.441 | (0.254) |
| 55 and above | 1.920** | (0.251) | 1.916* | (0.252) | 1.883* | (0.254) |
| South | 0.829 | (0.171) | 0.850 | (0.173) | 0.862 | (0.174) |
| Education (Omitted = High School or less) |  |  |  |  |  |  |
| Some college | 1.071 | (0.264) | 1.053 | (0.264) | 1.081 | (0.267) |
| College degree | 1.257 | (0.273) | 1.219 | (0.274) | 1.276 | (0.277) |
| More than college degree | 1.417 | (0.289) | 1.319 | (0.292) | 1.363 | (0.295) |
| Interaction with Non-Whites | 1.020 | (0.061) | 1.011 | (0.061) | 0.999 | (0.062) |
| Republican leaning | 0.996 | (0.017) | 1.002 | (0.017) | 1.002 | (0.017) |
| Constant | 2.640* | (0.397) | 3.966** | (0.480) | 5.567** | (0.495) |
|  |  | |  | |  | |
| -2 Log likelihood | 978.005 | | 975.648 | | 962.878 | |
| Nagelkerke R^2^ | 0.037 | | 0.040 | | 0.060 | |
| Notes: * p < 0.05; ** p < 0.01; *** p < 0.001.  ^a^ Cases are reduced because of missing observations on relative warmth toward Republican party.  Estimation method is logistic regression. Model 1 shows odds of remaining in sample, controlling for demographics and genetic knowledge. Model 2 additionally controls for pre-test genetic essentialism and Model 3 further controls for study arm allocation. | | | | | | |
