# Supplementary material for: Do genetic ancestry tests increase racial essentialism? Findings from a randomized controlled trial
Source: PLoS One. 2020 Jan 29;15(1):e0227399. doi: 10.1371/journal.pone.0227399 (PMC6988910; doi:10.1371/journal.pone.0227399)
Supplement: S2 Table — (DOCX) [file pone.0227399.s006.docx]

|  | **All Participants** | | **Participants Lost to Follow-Up** | | **Remaining Participants** | |
| --- | --- | --- | --- | --- | --- | --- |
|  | Control | Treatment | Control | Treatment | Control | Treatment |
| *Gender:* |  |  |  |  |  |  |
| Male | 36.50% | 35.80% | 35.10% | 33.60% | 36.70% | 36.60% |
| Female | 63.50% | 64.20% | 64.90% | 66.40% | 63.30% | 63.40% |
| *Age:* |  |  |  |  |  |  |
| 19-34 | 10.80% | 11.50% | 10.40% | 19.50% | 10.80% | 8.80% |
| 35-54 | 35.70% | 40.40% | 37.70% | 43.00% | 35.30% | 39.50% |
| 55 and above | 53.60% | 48.10% | 51.90% | 37.50% * | 53.90% | 51.70% |
| *Region:* |  |  |  |  |  |  |
| South | 30.50% | 33.10% | 35.10% | 39.10% | 29.60% | 31.00% |
| West | 24.50% | 21.60% | 20.80% | 18.80% | 25.20% | 22.50% |
| Midwest | 27.50% | 26.30% | 26.00% | 24.20% | 27.80% | 27.10% |
| Northeast | 17.50% | 19.00% | 18.20% | 18.00% | 17.40% | 19.40% |
| *Education:* |  |  |  |  |  |  |
| High school or less | 12.40% | 10.50% | 10.40% | 16.40% | 12.70% | 8.50% |
| Some college | 29.10% | 29.10% | 28.60% | 36.70% | 29.20% | 26.50% |
| College degree | 27.10% | 30.50% | 31.20% | 26.60% | 26.40% | 31.80% |
| More than college | 31.50% | 29.90% | 29.90% | 20.30% | 31.80% | 33.20% |
| *Genetic Knowledge:* |  |  |  |  |  |  |
| No Knowledge | 7.40% | 6.10% | 7.80% | 7.80% | 7.30% | 5.60% |
| Low Knowledge | 49.40% | 51.50% | 57.10% | 59.30% | 48.00% | 48.80% |
| Medium Knowledge | 11.40% | 10.70% | 7.80% | 9.40% | 12.00% | 11.10% |
| High Knowledge | 31.90% | 31.70% | 27.30% | 23.40% | 32.70% | 34.50% |
|  |  |  |  |  |  |  |
| Interaction with Non-Whites [1,7] | 3.31 (1.35) | 3.187 (1.371) | 3.478 (1.349) | 3.028 * (1.332) | 3.28 (1.349) | 3.24 (1.382) |
| Republican leaning [-10,10] | -0.996 (4.894) | -0.91 (5.093) | -0.506 (4.776) | -0.524 (4.705) | -1.086 (4.915) | -1.037 (5.215) |
| Pre-test Genetic Essentialism [0,1] | 0.457 (0.171) | 0.462 (0.168) | 0.492 (0.177) | 0.493 (0.162) | 0.467 (0.141) | 0.469 (0.141) |
| N | 502 | 505 | 77 | 128 | 425 | 377 |
| * p < 0.05; ** p < 0.01; *** p < 0.001  Note: Percentages are shown for categorical variables. The last three variables are continuous. Their range is given next to the variable name, and their means are presented, with the standard deviation in parentheses. The Pre-test Genetic Essentialism values for the Remaining Participants are estimated on the Remaining Participants sample. Our final analysis with the Remaining Participants used mixed-models, which required a long-shaped dataset with posttest data entered as separate cases. Rescaling of Pre-test Genetic Essentialism is done on this dataset, as opposed to the wide-shaped dataset with pretest and posttest data entered in the same respondent-level case. When the rescaling is done on the wide-shaped dataset, the mean values are .444 (.170) for Control and .445 (.169) for Treatment respectively. Difference in scaling has no effect on t-test coefficients. | | | | | | |
