# Supplementary material for: Do genetic ancestry tests increase racial essentialism? Findings from a randomized controlled trial
Source: PLoS One. 2020 Jan 29;15(1):e0227399. doi: 10.1371/journal.pone.0227399 (PMC6988910; doi:10.1371/journal.pone.0227399)
Supplement: S3 Table — (DOCX) [file pone.0227399.s007.docx]

|  | **Study Sample** | |  | **ACS Sample** | | **GKAP Sample** | |
| --- | --- | --- | --- | --- | --- | --- | --- |
|  | % | Freq. |  | % | Freq. | % | Freq. |
| *Gender* |  |  |  |  |  |  |  |
| Male | 36.7 | 294 |  | 48.8 | 786,177 |  |  |
| Female | 63.3 | 508 |  | 51.2 | 838,209 |  |  |
|  |  |  |  |  |  |  |  |
| *Age* |  |  |  |  |  |  |  |
| 19-34 | 9.9 | 79 |  | 25.3 | 350,543 |  |  |
| 35-54 | 37.3 | 299 |  | 32.5 | 499,654 |  |  |
| 55 and above | 52.9 | 424 |  | 42.2 | 780,836 |  |  |
|  |  |  |  |  |  |  |  |
| *Region* |  |  |  |  |  |  |  |
| South | 30.3 | 243 |  | 35.9 | 581,327 |  |  |
| West | 23.9 | 192 |  | 19.3 | 304,264 |  |  |
| Midwest | 27.4 | 220 |  | 26.4 | 431,792 |  |  |
| Northeast | 18.3 | 147 |  | 18.5 | 306,943 |  |  |
|  |  |  |  |  |  |  |  |
| *Education* |  |  |  |  |  |  |  |
| High school or less | 10.7 | 86 |  | 35.8 | 594,687 |  |  |
| Some college | 27.9 | 224 |  | 23.7 | 375,842 |  |  |
| College degree | 28.9 | 232 |  | 29.1 | 460.574 |  |  |
| More than college | 32.4 | 260 |  | 11.5 | 193,223 |  |  |
|  |  |  |  |  |  |  |  |
| *Genetic Knowledge Questions* | |  |  |  |  |  |  |
| Based on what you know, would you say that DNA can be found in every cell in the human body or only in specific organs and cells in the human body? | | | | | | | |
| In every cell | 92.3 | 740 |  |  |  | 76.7 | 879 |
| Only in specific organs and cells | 1.0 | 8 |  |  |  | 3.6 | 39 |
| Don’t know enough to say | 6.7 | 54 |  |  |  | 18.9 | 202 |
| Refused | **--** | **--** |  |  |  | 0.8 | 8 |
|  |  |  |  |  |  |  |  |
| Based on what you know, would you say that more than half, about half, or less than half of a human being’s genes are identical to those of a mouse? | | | | | | | |
| More than half | 34.5 | 277 |  |  |  | 19.5 | 238 |
| About half | 10.8 | 87 |  |  |  | 8.5 | 99 |
| Less than half | 16.3 | 131 |  |  |  | 19.3 | 209 |
| Don’t know enough to say | 38.3 | 307 |  |  |  | 51.4 | 571 |
| Refused | **--** | **--** |  |  |  | 1.2 | 11 |
|  |  |  |  |  |  |  |  |
| N |  | 802 |  |  | 1,624,326 |  | 1,128 |
| Notes: The study sample is our final analytical sample. The 2015 American Community Survey (ACS) sample is restricted to respondents who are native-born non-Hispanic White and aged 19 or older, approximately 51.6% of the total 2015 ACS sample. The Survey on Genomics Knowledge, Attitudes and Policy Views (GKAP) sample is restricted to respondents who are non-Hispanic White adults, approximately 26.3% of the total GKAP sample. ACS and GKAP data are weighted. | | | | | | | |
