# Supplementary material for: Do genetic ancestry tests increase racial essentialism? Findings from a randomized controlled trial
Source: PLoS One. 2020 Jan 29;15(1):e0227399. doi: 10.1371/journal.pone.0227399 (PMC6988910; doi:10.1371/journal.pone.0227399)
Supplement: S4 Table — (DOCX) [file pone.0227399.s008.docx]

| Prompt: “For the following statements about race and genetics, please check if you strongly agree, somewhat agree, somewhat disagree, or strongly disagree.” | |
| --- | --- |
| **Items** | **Statements** |
| Athlete | Certain races may be better athletes than others because of genetics. |
| Smart | Certain races may be smarter than others because of genetics. |
| Pure | There used to be “pure” races in the past. |
| PopDivide | The human population is divided into biological races. |
| GeneticsTells | No matter what a person looks like, genetics can tell what race they really are. |
| Classify | DNA technology will help us develop better racial classifications based on genetics. |
| RaceInvent [R] | Races are groups that societies invent. |
| AllAfrican [R] | Everyone’s ancestors originally came from Africa. |
| AllShare [R] | People of all races share most of the same genes. |

Notes: [R] = Reverse coded.
