# Supplementary material for: Do genetic ancestry tests increase racial essentialism? Findings from a randomized controlled trial
Source: PLoS One. 2020 Jan 29;15(1):e0227399. doi: 10.1371/journal.pone.0227399 (PMC6988910; doi:10.1371/journal.pone.0227399)
Supplement: S5 Table — (DOCX) [file pone.0227399.s009.docx]

| 1. Based on what you know, would you say that DNA can be found in every cell in the human body or only in specific organs and cells in the human body? | | |
| --- | --- | --- |
| **Response Choices** | **Frequency** | **Point Value** |
| In almost every cell in the human body | 91.86 | 1 |
| Only in specific organs and cells in the human body | 0.89 | 0 |
| Don't know enough to say | 7.25 | 0 |
|  |  |  |
| 1. Based on what you know, would you say that more than half, about half, or less than half of a human being’s genes are identical to those of a mouse? | | |
| **Response Choices** | **Frequency** | **Point Value** |
| More than half | 32.87 | 1 |
| About half | 10.23 | ½ |
| Less than half | 16.98 | 0 |
| Don’t know enough to say | 39.92 | 0 |
