# Supplementary material for: Do genetic ancestry tests increase racial essentialism? Findings from a randomized controlled trial
Source: PLoS One. 2020 Jan 29;15(1):e0227399. doi: 10.1371/journal.pone.0227399 (PMC6988910; doi:10.1371/journal.pone.0227399)
Supplement: S6 Table — (DOCX) [file pone.0227399.s010.docx]

| **Genetic Knowledge**  **raw scores** | Freq. | % |  | **Genetic Knowledge Ordinal Categories** | Freq. | % |  | **Genetic Knowledge Dichotomous Categories** | Freq. | % |
| --- | --- | --- | --- | --- | --- | --- | --- | --- | --- | --- |
|  |  |  |  |  |  |  |  |  |  |  |
| 0 = 0.000 | 52 | 6.48 |  | No Knowledge (0) | 52 | 6.48 |  | Lower Knowledge (0,1,2) | 440 | 54.86 |
| 1 = 0.011 | 386 | 48.13 |  | Low Knowledge (1,2) | 388 | 48.38 |  | Higher Knowledge (3,4,5) | 362 | 45.14 |
| 2 = 0.013 | 2 | 0.25 |  | Medium Knowledge (3,4) | 93 | 11.6 |  |  |  |  |
| 3 = 0.024 | 85 | 10.6 |  | High Knowledge (5) | 269 | 33.54 |  |  |  |  |
| 4 = 0.026 | 8 | 1 |  |  |  |  |  |  |  |  |
| 5 = 0.037 | 269 | 33.54 |  |  |  |  |  |  |  |  |
|  |  |  |  |  |  |  |  |  |  |  |
| Total | 802 | 100 |  | Total | 802 | 100 |  | Total | 802 | 100 |
