# Supplementary material for: Do genetic ancestry tests increase racial essentialism? Findings from a randomized controlled trial
Source: PLoS One. 2020 Jan 29;15(1):e0227399. doi: 10.1371/journal.pone.0227399 (PMC6988910; doi:10.1371/journal.pone.0227399)
Supplement: S7 Table — (DOCX) [file pone.0227399.s011.docx]

|  | N | % |
| --- | --- | --- |
| Listed a specific European ancestry | 362 | 96.0 |
| - Any known European ancestry was “confirmed” | 348 | 92.3 |
| - Any known European ancestry was not “confirmed” | 16 | 4.2 |
|  |  |  |
| Listed a specific non-European ancestry | 60 | 15.9 |
| - Any known non-European ancestry was “confirmed” | 4 | 1.1 |
| - Any known non-European ancestry was not “confirmed” | 56 | 14.9 |
|  |  |  |
| “Discovered” a new European ancestry from tests | 343 | 91.0 |
| “Discovered” a new non-European ancestry from tests | 230 | 61.0 |
| Notes: European ancestry includes “Ashkenazi Jewish.” Respondents who did not list a specific European ancestry typically wrote in only broad terms like “White” or “European”. “Confirmed” and “Discovered” are in quotation marks to reflect that tests reporting known or new ancestries felt like confirmation or discovery to many respondents, but the test results are subject to limitations and these interpretations may be incorrect. | | |
