# Supplementary material for: Do genetic ancestry tests increase racial essentialism? Findings from a randomized controlled trial
Source: PLoS One. 2020 Jan 29;15(1):e0227399. doi: 10.1371/journal.pone.0227399 (PMC6988910; doi:10.1371/journal.pone.0227399)
Supplement: S8 Table — (DOCX) [file pone.0227399.s012.docx]

| Genetic Essentialism for Race | Mean | (S.D.) |
| --- | --- | --- |
| **Treatment Group** | -0.021 | (0.017) |
| **Post-Test** | -0.015 | (0.010) |
| **Treatment Group x Post-Test** | -0.025 | (0.051) |
| **Genetic Knowledge** (Omitted=High) |  |  |
| No Knowledge | 0.056 | (0.029) |
| Low Knowledge | 0.043** | (0.016) |
| Medium Knowledge | 0.009 | (0.023) |
| **Genetic Knowledge x Treatment Group** |  |  |
| No Knowledge | 0.017 | (0.044) |
| Low Knowledge | 0.032 | (0.023) |
| Medium Knowledge | 0.061 | (0.034) |
| **Genetic Knowledge x Post-Test** |  |  |
| No Knowledge | 0.028 | (0.024) |
| Low Knowledge | 0.032* | (0.013) |
| Medium Knowledge | 0.019 | (0.020) |
| **Treatment x Post-Test x Genetic Knowledge** |  |  |
| No Knowledge | 0.070 | (0.037) |
| Low Knowledge | 0.011 | (0.019) |
| Medium Knowledge | 0.041 | (0.029) |
| **South** | 0.022* | (0.010) |
| **Interaction with Non-Whites** | -0.008* | (0.003) |
| **Republican leaning** | 0.008*** | (0.001) |
| **Male** (Omitted = Female) | -0.010 | (0.010) |
| **Age** (Omitted= 19-34) |  |  |
| 35-54 | 0.017 | (0.017) |
| 55 above | -0.000 | (0.016) |
| **Education** (Omitted=High School or less) |  |  |
| Some college | -0.031 | (0.017) |
| College degree | -0.040* | (0.017) |
| More than a college degree | -0.088*** | (0.017) |
| Constant | 0.517*** | (0.026) |
| **Random Effects Parameters** |  |  |
| Between-Subject Variance | 0.0128 | (0.001) |
| Within-Subject Variance | 0.0073 | (0.000) |
| Observations | 1588 | |
| Number of Groups | 794 | |

* p < 0.05; ** p < 0.01; *** p < 0.001
