# Supplementary material for: Do genetic ancestry tests increase racial essentialism? Findings from a randomized controlled trial
Source: PLoS One. 2020 Jan 29;15(1):e0227399. doi: 10.1371/journal.pone.0227399 (PMC6988910; doi:10.1371/journal.pone.0227399)
Supplement: S9 Table — (DOCX) [file pone.0227399.s013.docx]

|  | Contrast | Std. Error | P>\|z\| | Conf. Interval | | N |
| --- | --- | --- | --- | --- | --- | --- |
| **Post-test vs. Pre-test** |  |  |  |  |  |  |
| **No Knowledge** |  |  |  |  |  |  |
| Control | 0.013 | 0.022 | 0.543 | -0.029 | 0.055 | 31 |
| Treatment | 0.058 | 0.026 | 0.026 | 0.007 | 0.110 | 21 |
| **Low Knowledge** |  |  |  |  |  |  |
| Control | 0.018 | 0.009 | 0.035 | 0.001 | 0.035 | 200 |
| Treatment | 0.004 | 0.009 | 0.667 | -0.014 | 0.021 | 183 |
| **Medium Knowledge** |  |  |  |  |  |  |
| Control | 0.004 | 0.017 | 0.804 | -0.029 | 0.038 | 50 |
| Treatment | 0.020 | 0.019 | 0.277 | -0.016 | 0.057 | 42 |
| **High Knowledge** |  |  |  |  |  |  |
| Control | -0.015 | 0.010 | 0.157 | -0.035 | 0.006 | 138 |
| Treatment | -0.040 | 0.011 | 0.000 | -0.060 | -0.019 | 129 |

Note: Each row indicates contrast of the respective group in terms of their score in post-test vs. pre-test. Confidence intervals are at 95% level.
