# Supplementary material for: Do genetic ancestry tests increase racial essentialism? Findings from a randomized controlled trial
Source: PLoS One. 2020 Jan 29;15(1):e0227399. doi: 10.1371/journal.pone.0227399 (PMC6988910; doi:10.1371/journal.pone.0227399)
Supplement: S10 Table — (DOCX) [file pone.0227399.s014.docx]

|  | **Model 1** | | **Model 2** | | **Model 3** | |
| --- | --- | --- | --- | --- | --- | --- |
|  | Coef. | (S.E.) | Coef. | (S.E.) | Coef. | (S.E.) |
| **Pre-Test Genetic Essentialism** | 0.863*** | (0.034) | 0.828*** | (0.034) | 0.824*** | (0.034) |
| **Treatment Group** | -0.011 | (0.009) | -0.010 | (0.008) | -0.032* | (0.015) |
| **Genetic Knowledge** (Omitted = High) |  |  |  |  |  |  |
| No Knowledge |  |  | 0.066*** | (0.019) | 0.029 | (0.024) |
| Low Knowledge |  |  | 0.046*** | (0.010) | 0.035** | (0.014) |
| Medium Knowledge |  |  | 0.044** | (0.014) | 0.020 | (0.020) |
| **Treatment x Genetic Knowledge** |  |  |  |  |  |  |
| Treatment x No Knowledge |  |  |  |  | 0.086* | (0.037) |
| Treatment x Low Knowledge |  |  |  |  | 0.022 | (0.019) |
| Treatment x Medium Knowledge |  |  |  |  | 0.052 | (0.029) |
| **South** | -0.001 | (0.009) | -0.002 | (0.009) | -0.003 | (0.009) |
| **Interactions with Non-Whites** | -0.001 | (0.003) | 0.000 | (0.003) | 0.000 | (0.003) |
| **Republican leaning** | 0.003*** | (0.001) | 0.003*** | (0.001) | 0.003*** | (0.001) |
| **Male** (Omitted = Female) | -0.011 | (0.009) | -0.009 | (0.009) | -0.009 | (0.009) |
| **Age** (Omitted = 19-34) |  |  |  |  |  |  |
| 35-54 | 0.007 | (0.016) | 0.008 | (0.015) | 0.007 | (0.015) |
| 55 above | 0.003 | (0.02) | -0.000 | (0.015) | -0.000 | (0.015) |
| **Education (**Omitted = HS or less) |  |  |  |  |  |  |
| Some college | -0.027 | (0.016) | -0.023 | (0.015) | -0.025 | (0.015) |
| College degree | -0.021 | (0.016) | -0.014 | (0.016) | -0.017 | (0.016) |
| More than a college degree | -0.035* | (0.016) | -0.024 | (0.016) | -0.027 | (0.016) |
| Constant | 0.101*** | (0.028) | 0.076** | (0.028) | 0.093** | (0.029) |
| Adjusted *R*^2^ | 0.544 | | 0.558 | | 0.560 | |
| Notes: * p < 0.05; ** p < 0.01; *** p < 0.001.  For reference category of the Genetic Knowledge Variable, we used High Knowledge because interpretation is more sensible, and it has around 33% of the observations for both the control and the treatment groups. Estimation method is Ordinary Least Squares. Model 1 is the base model, showing the effect of Pre-test Genetic Essentialism and assignment to the Treatment Group without the Genetic Knowledge variable while controlling for demographic, social, and political variables. Model 2, building on the base model, adds Genetic Knowledge. Model 3 shows the interaction of Genetic Knowledge with the Group variable. | | | | | | |
