# Supplementary material for: Do genetic ancestry tests increase racial essentialism? Findings from a randomized controlled trial
Source: PLoS One. 2020 Jan 29;15(1):e0227399. doi: 10.1371/journal.pone.0227399 (PMC6988910; doi:10.1371/journal.pone.0227399)
Supplement: S11 Table — (DOCX) [file pone.0227399.s015.docx]

|  | Contrast | Std. Error | P>\|z\| | Conf. Interval | | N |
| --- | --- | --- | --- | --- | --- | --- |
| **Post-test vs. Pre-test** |  |  |  |  |  |  |
| **Lower Knowledge** |  |  |  |  |  |  |
| Control | 0.017 | 0.008 | 0.030 | 0.002 | 0.033 | 231 |
| Treatment | 0.009 | 0.008 | 0.265 | -0.007 | 0.026 | 204 |
| **Higher Knowledge** |  |  |  |  |  |  |
| Control | -0.010 | 0.009 | 0.280 | -0.027 | 0.008 | 188 |
| Treatment | -0.025 | 0.009 | 0.007 | -0.043 | -0.007 | 171 |

Note: Each row indicates contrast of the respective group in terms of their score in post-test vs. pre-test. Lower genetic knowledge combines ‘no’ and ‘low’ knowledge. Higher genetic knowledge combines ‘medium’ and ‘high’ knowledge. Confidence intervals are at 95% level.
