# Supplementary material for: Do genetic ancestry tests increase racial essentialism? Findings from a randomized controlled trial
Source: PLoS One. 2020 Jan 29;15(1):e0227399. doi: 10.1371/journal.pone.0227399 (PMC6988910; doi:10.1371/journal.pone.0227399)
Supplement: S12 Table — (DOCX) [file pone.0227399.s016.docx]

|  | **Model 1** | | **Model 2** | | **Model 3** | |
| --- | --- | --- | --- | --- | --- | --- |
|  | Coef. | (SE) | Coef. | (SE) | Coef. | (SE) |
| **Pre-Test Genetic Essentialism** | 0.821*** | (0.053) | 0.747*** | (0.053) | 0.748*** | (0.053) |
| **European Ancestry “Confirmed”** | -0.054 | (0.035) | -0.056 | (0.033) | -0.046 | (0.051) |
| **Male (Omitted = Female)** | -0.011 | (0.014) | -0.006 | (0.013) | -0.006 | (0.014) |
| **Age (Omitted = between 19-34 )** |  |  |  |  |  |  |
| between 35-54 | 0.029 | (0.026) | 0.028 | (0.025) | 0.027 | (0.025) |
| 55 above | 0.018 | (0.025) | 0.005 | (0.024) | 0.005 | (0.024) |
| **Education (Omitted = HS or less)** |  |  |  |  |  |  |
| Some college | -0.045 | (0.027) | -0.052* | (0.026) | -0.052* | (0.026) |
| College degree | -0.053 | (0.027) | -0.061* | (0.026) | -0.061* | (0.027) |
| More than a college degree | -0.039 | (0.028) | -0.037 | (0.027) | -0.037 | (0.027) |
| **Interactions with Non-Whites** | -0.002 | (0.004) | -0.001 | (0.004) | -0.001 | (0.004) |
| **Republican leaning** | 0.004** | (0.001) | 0.005*** | (0.001) | 0.005*** | (0.001) |
| **South** | -0.009 | (0.015) | -0.012 | (0.014) | -0.012 | (0.014) |
| **Genetic Knowledge (Omitted = High)** |  |  |  |  |  |  |
| No Knowledge |  |  | 0.122*** | (0.031) | 0.103 | (0.132) |
| Low Knowledge |  |  | 0.068*** | (0.015) | 0.097 | (0.070) |
| Medium Knowledge |  |  | 0.077*** | (0.022) | 0.061 | (0.132) |
| **“Confirmed” Eur. ancestry x Genetic Knowledge** |  |  |  |  |  |  |
| “Confirmed” Eur. ancestry x No  Knowledge |  |  |  |  | 0.020 | (0.135) |
| “Confirmed” Eur. ancestry x Low  Knowledge |  |  |  |  | -0.030 | (0.072) |
| “Confirmed” Eur. ancestry x Med.  Knowledge |  |  |  |  | 0.016 | (0.134) |
| Constant | 0.167** | (0.058) | 0.162** | (0.055) | 0.152* | (0.067) |
| Adjusted *R*^2^ | 0.502 | | 0.538 | | 0.535 | |
| * p< .05; ** p<.01; *** p<.001. N=360 |  |  |  |  |  |  |
