# Supplementary material for: Do genetic ancestry tests increase racial essentialism? Findings from a randomized controlled trial
Source: PLoS One. 2020 Jan 29;15(1):e0227399. doi: 10.1371/journal.pone.0227399 (PMC6988910; doi:10.1371/journal.pone.0227399)
Supplement: S13 Table — (DOCX) [file pone.0227399.s017.docx]

|  | **Model 1** | | **Model 2** | | **Model 3** | |
| --- | --- | --- | --- | --- | --- | --- |
|  | Coef. | (SE) | Coef. | (SE) | Coef. | (SE) |
| **Pre-Test Genetic Essentialism** | 0.828*** | (0.159) | 0.815*** | (0.162) | 0.813*** | (0.164) |
| **Non-European Ancestry “Confirmed”** | -0.202** | (0.073) | -0.208** | (0.075) | -0.241 | (0.145) |
| **Male (Omitted=Female)** | 0.043 | (0.043) | 0.049 | (0.044) | 0.047 | (0.045) |
| **Age (Omitted=between 19-34 )** |  |  |  |  |  |  |
| between 35-54 | 0.083 | (0.056) | 0.081 | (0.057) | 0.080 | (0.058) |
| 55 above | 0.039 | (0.062) | 0.025 | (0.064) | 0.024 | (0.065) |
| **Education (Omitted=High school or less)** |  |  |  |  |  |  |
| Some college | 0.027 | (0.060) | 0.027 | (0.061) | 0.026 | (0.062) |
| College degree | 0.020 | (0.068) | 0.026 | (0.070) | 0.023 | (0.071) |
| More than a college degree | 0.024 | (0.074) | 0.052 | (0.080) | 0.053 | (0.081) |
| **Interactions with Non-Whites** | -0.020 | (0.013) | -0.023 | (0.013) | -0.022 | (0.013) |
| **Republican leaning** | 0.002 | (0.003) | 0.002 | (0.003) | 0.002 | (0.003) |
| **South** | -0.022 | (0.040) | -0.032 | (0.042) | -0.033 | (0.043) |
| **Genetic Knowledge (Omitted=High)** |  |  |  |  |  |  |
| No Knowledge |  |  | 0.069 | (0.089) | 0.069 | (0.090) |
| Low Knowledge |  |  | 0.053 | (0.044) | 0.051 | (0.045) |
| Medium Knowledge |  |  | 0.026 | (0.065) | 0.023 | (0.066) |
| **“Confirmed” Non-Eur. ancestry x Genetic Knowledge** |  |  |  |  |  |  |
| “Confirmed” Non-Eur. ancestry x  No Knowledge |  |  |  |  | 0.000 | . |
| “Confirmed” Non-Eur. ancestry x  Low Knowledge |  |  |  |  | 0.046 | (0.171) |
| “Confirmed” Non-Eur. ancestry x  Med. Knowledge |  |  |  |  | 0.000 | . |
| Constant | 0.099 | (0.125) | 0.084 | (0.128) | 0.089 | (0.130) |
| Adjusted *R*^2^ | 0.489 | | 0.473 | | 0.462 | |
| * p < 0.05; ** p < 0.01; *** p < 0.001. N=60 |  |  |  |  |  |  |
