# Supplementary material for: Do genetic ancestry tests increase racial essentialism? Findings from a randomized controlled trial
Source: PLoS One. 2020 Jan 29;15(1):e0227399. doi: 10.1371/journal.pone.0227399 (PMC6988910; doi:10.1371/journal.pone.0227399)
Supplement: S14 Table — (DOCX) [file pone.0227399.s018.docx]

|  | **Model 1** | | **Model 2** | | **Model 3** | |
| --- | --- | --- | --- | --- | --- | --- |
|  | Coef. | (SE) | Coef. | (SE) | Coef. | (SE) |
| **Pre-Test Genetic Essentialism** | 0.758*** | (0.05) | 0.756*** | (0.05) | 0.759*** | (0.05) |
| **“Discovery” of Non-European Ancestry** | -0.002 | (0.01) | -0.001 | (0.01) | 0.013 | (0.02) |
| **“Discovery” of European Ancestry** | 0.014 | (0.02) | 0.020 | (0.04) | 0.015 | (0.02) |
| **Male (Omitted=Female)** | -0.003 | (0.01) | -0.004 | (0.01) | -0.004 | (0.01) |
| **Age (Omitted=between 19-34 )** |  |  |  |  |  |  |
| between 35-54 | 0.023 | (0.02) | 0.022 | (0.02) | 0.017 | (0.02) |
| 55 above | -0.001 | (0.02) | -0.001 | (0.02) | -0.006 | (0.02) |
| **Education (Omitted=High school or less)** |  |  |  |  |  |  |
| Some college | -0.042 | (0.02) | -0.044 | (0.03) | -0.050 | (0.03) |
| College degree | -0.046 | (0.02) | -0.047 | (0.03) | -0.052* | (0.03) |
| More than a college degree | -0.026 | (0.03) | -0.027 | (0.03) | -0.031 | (0.03) |
| **Interactions with Non-Whites** | -0.002 | (0.00) | -0.002 | (0.00) | -0.002 | (0.00) |
| **Republican leaning** | 0.005*** | (0.00) | 0.005*** | (0.00) | 0.005*** | (0.00) |
| **South** | -0.008 | (0.01) | -0.007 | (0.01) | -0.009 | (0.01) |
| **Genetic Knowledge (Omitted=High)** |  |  |  |  |  |  |
| No Knowledge | 0.127*** | (0.03) | 0.245 | (0.13) | 0.167*** | (0.04) |
| Low Knowledge | 0.068*** | (0.01) | 0.065 | (0.05) | 0.084*** | (0.02) |
| Medium Knowledge | 0.077*** | (0.02) | 0.099 | (0.06) | 0.065 | (0.03) |
| **“Discovery” of Eur. Ancestry x Genetic**  **Knowledge** |  |  |  |  |  |  |
| “Discovery of Eur. Ancestry x No Knowledge |  |  | -0.124 | (0.13) |  |  |
| “Discovery” of Eur. Ancestry x Low Knowledge |  |  | 0.003 | (0.05) |  |  |
| “Discovery” of Eur. Ancestry x Med. Knowledge |  |  | -0.025 | (0.07) |  |  |
| **“Discovery” of Non-Eur. Ancestry x Genetic**  **Knowledge** |  |  |  |  |  |  |
| “Discovery” of non-Eur. Ancestry x No  Knowledge |  |  |  |  | -0.086 | (0.06) |
| “Discovery” of non-Eur. Ancestry x Low  Knowledge |  |  |  |  | -0.025 | (0.03) |
| “Discovery” of non-Eur. Ancestry x Medium  Knowledge |  |  |  |  | 0.023 | (0.04) |
| Constant | 0.088 | (0.05) | 0.085 | (0.06) | 0.090 | (0.05) |
| Adjusted *R*^2^ | 0.549 | | 0.547 | | 0.550 | |
| * p< .05; ** p<.01; *** p<.001. N=375 |  |  |  |  |  |  |
