# Supplementary material for: Do genetic ancestry tests increase racial essentialism? Findings from a randomized controlled trial
Source: PLoS One. 2020 Jan 29;15(1):e0227399. doi: 10.1371/journal.pone.0227399 (PMC6988910; doi:10.1371/journal.pone.0227399)
Supplement: Attachment — Submitted filename: PLOSreview.docx [file pone.0227399.s019.docx]

This paper asks an important question regarding the way in which the increasing availability of genetic ancestry testing may influence the degree of people’s racial essentialism.  The study employs a sound pre and post-test experimental procedure to test the question.  The authors’ review of literature and rationale for the research question is clear and to the point.  The paper becomes muddy in the results section and I have questions about the strength of the moderator finding, and the analytic approach used to test the moderation.  I suspect the authors’ chose this approach because it results in some arguable significance in their findings; and, I suspect a more appropriate analysis would result in a null finding.  I am not at all suggesting any academic dishonesty by the authors; I certainly understand the pressure we as researchers are under to arrive at significance in order to have our good research questions taken seriously.  My preference however, would be to see a cleaner analysis even if it does not result in significance.  In turn, I would argue that it is our responsibility as reviewers and editors to appreciate the contribution of the article based on the merits of the question and rationale.  In other words, if it were my choice, I would accept this article on the basis of the question. Below, I discuss my concerns about the analysis—which is my only major concern about the paper—and then list a number of minor edits or changes I would like to see made.

**Major Concern**

The authors’ find that there is no main effect of those exposed to ancestry testing (compared to those who are not) on the racial essentialism outcome. The authors’ hypothesize that people’s genetic literacy may moderate these findings (i.e., is working as a suppressor variable). In order to test the moderation, the researchers created four groups: high, medium, low, and no knowledge groups.

From table 8, it appears that the authors tested interactions by comparing the no, low, and medium groups to the high knowledge group, as three separate tests of moderation. The authors’ find that there is an interaction effect but only between the “no knowledge” and “high knowledge groups.” I am concerned about the strength of this findings because it is really only based on about half the sample as both low and medium knowledge groups are not included. Moreover, despite a large sample size and the multiple analyses (e.g., three interaction terms) this is significant only at .05. The creation of four groups appears convenient to the arrival of a significant finding, and does not appear to me to be the most intuitive or appropriate approach to test the moderation. I would be more convinced if the finding emerged based on either a dichotomized or continuous moderator, because in both cases all of the data would be contributing to the findings. However, I suspect if the researchers dichotomized this variable (into low and high genetic literacy) it would not reach significance. The genetic literacy variable could be treated as a continuous variable (as it is merely the number of questions answered correctly); again, I would be more convinced of the finding if there was a single analysis with genetic literacy entered as a continuous moderator (the PROCESS macro is SPSS is capable of running continuous moderators). Perhaps if no finding emerges related to either the dichotomized or continuous variable moderation, then the authors could run the four-group approach as a post-hoc, but would need to talk candidly about the limits of this approach in the discussion.

Related to this concern, on page 10, line 224 the authors mention the use of OLS to *confirm* moderation, but I don’t see a previous moderation finding to be confirmed. In the text the authors state that there are significant decreases in the treatment condition, but not significant decreases in the control; and while this certainly resembles a moderation, there is no statistic provided (aside from the OLS) to determine that this is a significant moderator.

**Minor Concerns**

- On page 3 line 46, specify “affects” (e.g., “…affects users’ beliefs about race and consequently, their intergroup behaviors”)
- On page 3 line 52, specify that “This” refers to genetic essentialism
- On page 3 line 52; The authors state that this is “one type of essentialism” which—in conjunction with their description of the three factor measure—seems to be setting up the expectation that the impact of genetic testing on multiple types of essentialism will be examined.
- On page 6 line 123, “tests’” should be changed to “test’s”
- On page 8, line 177- 178 the authors’ claim that ”models that included this item showed similar results…” If this is the case, then it should include citations. Furthermore, I think the authors need to clarify then what is meant by similar results since in the literature review it was argued that this analysis hasn’t been done before (testing the effects of exposure to genetic ancestry on racial essentialism). It also occurs to me that perhaps it is meant that the authors also ran the model with this item included? If this is the case then the authors should clarify “models that *we* ran with this item included…” and perhaps the statistics could be included in a footnote.
- On page 9, line 204, add “…A non-experimental”
- On page 10 line 207 confirm that both models (LME and non-experimental test) included all the control variables
- What statistics procedure is used for the second hypothesis? (i.e., testing the effects of specific test results on genetic essentialism controlling for genetic literacy?)
- What statistic is being reported on page 10, lines 216, 217, 218, 220, 221, 223..
- Effect sizes should be reported throughout the results section.
- On page 10 lines 227 – 232 the authors describe changes in essentialism scores. I’m not sure this is necessary if it is in the figure. If the authors decide to keep it in the text, it should be more clear what the values are. Are these adjusted means? They don’t appear to be raw means based on description of the measure (9 items, 4 pt Likert scale)
- The authors make a distinction between three subscales but only do analyses on the full scale. Why?
- Some psychometrics should be provided for the essentialism scale. I see the authors reference another paper which presumably supports the psychometrics of the scale with CFA and EFA. The authors should then provide at a minimum Cronbach alpha coefficients for this scale with this current sample.
- The idea that specific results on genetic tests would be examined as an influence on racial essentialism should be introduced earlier in the paper (the first we hear of it is page 6 line 115)
- I am confused on page 6 line 118 about the direction of the hypothesis. I expected that discovery of new ancestry would relate to *less* essentialism, so perhaps specify if it is more or less? Or are you predicting a null main effect but a suppression effect of genetic literacy?
