# Supplementary material for: Do genetic ancestry tests increase racial essentialism? Findings from a randomized controlled trial
Source: PLoS One. 2020 Jan 29;15(1):e0227399. doi: 10.1371/journal.pone.0227399 (PMC6988910; doi:10.1371/journal.pone.0227399)
Supplement: Attachment — Submitted filename: Response to Reviewers.docx [file pone.0227399.s020.docx]

**Revision Memo**

**Manuscript Number: PONE-D-19-21371**

**Revised Title: “Do genetic ancestry tests increase racial essentialism? Findings from a randomized controlled trial”**

We would like to begin by sincerely thanking the reviewers for their detailed and helpful comments. The reviewers offered valid critiques, which have led us to rethink and revise many aspects of the paper. We feel that the paper is much improved as a result of the reviewers’ comments, and are very grateful for the time and effort that they put in to helping us improve it.

We are also extremely grateful for the many positive and encouraging comments from all the reviewers. We truly appreciate that you have found value in the paper and that you took the time to express it.

Below we address the comments of the reviewers and the Editor. We have grouped the comments thematically. The line numbers refer to the version with track changes removed.

**Genetic Knowledge as a Modifying Effect**

Reviewers 1 and 3 raised concerns about the presentation of the analysis of genetic knowledge. Reviewer 1 noted that because we did not manipulate genetic knowledge experimentally, we should emphasize that these findings are suggestive and they should be presented more cautiously. Similarly, Reviewer 3 cautioned against describing our findings as a moderation. In line with the reviewers’ suggestions, we have reframed the paper to distinguish between our primary analysis, based on the randomization to treatment and control groups, and the secondary analyses, which are not. We have changed the title of the paper accordingly, and have reorganized the description of the analysis in the introduction as well as the hypotheses to sequentially describe primary and secondary analyses. We had previously used the term ‘moderation’, intending it as a synonym for the effect we observed in our interaction models in the Ordinary Least Squares (OLS) regression, and did not mean to imply that we had conducted a formal moderation analysis. To clarify this, we have reworded our language to avoid readers interpreting it as a moderation analysis. We also adjusted the wording of the secondary analyses to remove causal language, and edited the discussion section to avoid implying that the findings surrounding genetic knowledge are causally related to the testing intervention.

Reviewer 3 also expressed concern about the structure of the genetic knowledge variable and the analytic approach used to test interactions between genetic knowledge and the study arm allocation group variable, particularly the comparison of the no, low, and medium knowledge groups to the ‘high knowledge’ group. The reviewer was concerned about the strength of the effect observed for the ‘no knowledge’ group as compared to ‘high knowledge’ group in the interaction model because the finding, the reviewer suggested, is based on about half the sample and because it is only significant at the .05 level. We appreciate the reviewer raising these concerns because it forced us to think carefully about this issue. First, we would like to point out that this comment seems to focus only on the OLS analysis (now S10 Table), which uses interactions between the genetic knowledge groups and assignment to the treatment group. However, we include the OLS analysis to lend further support to the mixed-effects models (S8 Table) and the post-estimation predictions that are based on them as presented in the pairwise contrasts (S9 Table). In particular, the pairwise contrasts show the predicted average probabilities of change in genetic essentialism within each genetic knowledge group between the pre-test and post-test surveys. Here, too, we see that the average predicted probability of treatment respondents in the ‘no knowledge’ group declined significantly between the pre-test and the post-test, and this is not simply relative to the ‘high knowledge’ group.

However, we acknowledge and agree with the reviewer that the finding is still based on a small number of cases in the ‘no knowledge’ group, and we have made several changes to be clearer about this and to test the robustness of our findings. As Reviewer 3 suggested, we did run the analyses with a dichotomized version of the genetic knowledge variable. We combined the ‘no/low’ knowledge categories and the ‘medium/high’ knowledge categories to create a binary measure of ‘lower’ and ‘higher’ genetic knowledge. With this binary measure, we find that the average predicted probabilities of genetic essentialism declined significantly in the treatment group with higher genetic knowledge (Δ = -0.025; p=0.007), while control respondents with higher genetic knowledge did not show a significant change (Δ = -0.010; p=0.280) (S11 Table and S3 Fig). This is in line with what we had found with the 4-category ordinal measure of genetic essentialism variable. We do not find a significant predicted change among treatment respondents with lower knowledge (Δ = 0.009; p=0.265), however. Thus, the dichotomous variable shows that our findings that high(er) genetic knowledge is predicted to decrease genetic essentialism after testing is robust; as expected, this analysis does not support the predicted increase in genetic essentialism among those with the least genetic knowledge because the ‘lower knowledge’ category is numerically dominated by those in the 4-category ‘low knowledge’ group.

The reviewer also suggested using a continuous version of the genetic knowledge variable; however, we do not think a continuous version of this variable would be viable given that there are only four effective categories out of the six in the full range, as two of the categories have very few cases in them (S6 Table). When categories are few, it is not recommended to treat a categorical variable as continuous; the recommendation is to have at least 5 and preferably 7 categories to treat an ordinal variable as a continuous variable (Liddell and Krusche 2018). Furthermore, the variable is not normally distributed (see S6 Table). Also given that we are now aware of potential differences in our results based on different genetic knowledge levels, we do not think it is reasonable to assume that the one-unit effect will be the same across the different points of a continuous scale.

We decided to include the analyses with both the 4-category ordinal and the dichotomous genetic knowledge variables in the paper. We believe that the preliminary indication of an expected increase in genetic essentialism for test-takers with no genetic knowledge is a noteworthy pattern that deserves further exploration. Our limitation is statistical power; in fact, it is noteworthy that we see a significant association despite the lack of statistical power. Because this is the first RCT of genetic ancestry testing on genetic essentialism (to our knowledge), we believe it is valuable to present the indication of a possible increase in genetic essentialism among those with the least genetic knowledge as something that might be interesting to explore in future research, while still using caution to avoid suggesting that this is a conclusive finding. We have edited our discussion to reflect this (lines 230-237; 257-258; 280-318; 369-373; 399-410). We have also added more information about these considerations in the supplement under the ‘Genetic Knowledge Variables’ section (p.5).

**Representativeness of Sample**

Reviewer 2 asked how representative the study population is of the U.S. non-Hispanic White population and said “The authors describe this as their goal during the study sampling, but never present information regarding how successful their sampling strategy was.” We apologize if our language was misleading, but want to clarify that the representativeness of the study population was not our goal. Our target population is not all native-born White non-Hispanics, but specifically native-born White non-Hispanics who are willing to take genetic ancestry tests (GATs) and have never received personalized genetic ancestry information. We expect this population to differ from the overall population of native-born White non-Hispanics, yet we were unable to find a representative sample of this particular target population before the study began. We have clarified this in the manuscript (lines 103-104; 150-152; 155-162). Nevertheless, we take the reviewer’s point that it is valuable for readers to know how this sample compares with the larger population of native-born White non-Hispanics. We have therefore added a table to the supplement (S3 Table) that compares our study sample to a representative sample of native-born White non-Hispanics from the 2015 American Community Survey. In the supplement (p.3-4), we discuss differences between our sample and this broader population, and note that these differences likely reflect patterns in who is willing to take genetic ancestry tests, as well as trends in who is willing to participate in research studies.

Reviewer 2 also asked how our sample’s knowledge of genetics compared to the larger general population. We therefore also added to S3 Table the responses to the survey items from the subsample of native-born White non-Hispanics in the Survey on Genomics Knowledge, Attitudes and Policy Views (GKAP). Again we compare our sample to this population, and note that those who are willing to take genetic ancestry tests (our target population) likely has higher genetic knowledge than all native-born White non-Hispanics.

**Study Design**

Reviewer 2 asked us to comment on why we chose to give our control group respondents no stimulus rather than some type of non-ancestry genetic test results. We made this decision because our goal is to simulate the effect of taking GATs relative to those who do not (as we now state on lines 106-107). We are interested in the potential effects of GATs on the population that takes them, rather than the relative effect of GATs vs. other genetic tests. To have compared those who take GATs to those who take health-based genetic tests, for instance, would be similar to more conventional non-inferiority clinical RCTs that test the equivalence of two treatments – whether one is inferior to the other for the treatment of a disease outcome. We expect that most people who do not take GATs likely do not take other genetic tests either. Therefore, we have maintained our control group based on the exploratory, innovative nature of the study and the fact that this type of RCT has not been conducted before (to our knowledge). Comparing the relative effect of different tests would be a reasonable next step after establishing the effect that concerns us here.

**Measures**

Both Reviewers 2 and 3 asked for additional information about the essentialism scale. We added in more information about the development and validity of the scale. We also added in the Cronbach's alpha for this sample, and provided a clearer reference to the publication where the scale's development and validation are described in detail (lines 190-208).

Reviewer 2 noted that our models adjust for living in the South and asked how geographic region was defined. We use the same definition as the U.S. Census Bureau statistical regions. Specifically, the South census region includes Alabama, Arkansas, Delaware, the District of Columbia, Florida, Georgia, Kentucky, Louisiana, Maryland, Mississippi, North Carolina, Oklahoma, South Carolina, Tennessee, Texas, Virginia, and West Virginia. We added a description for how the geographic region 'South' was defined to the Supplement, under 'Control Variables' (pp.6-7).

Reviewer 2 also asked us why we developed the genetic knowledge scale out of the two questions from the GKAP survey rather than using validated surveys for genetic knowledge like the one developed by Jallinoja and Aro. Our answer is that when we began this study, we did not expect genetic knowledge to emerge as a major focus and predicted that educational attainment would capture the differences in respondents’ understandings of their GAT results. We view this as a limitation of the study, and believe that future studies need to build on this work by using a more robust scale. Although we had already mentioned this as a limitation of the study in our discussion section, we have further emphasized this point and have specifically mentioned the Jallinoja and Aro scale to direct potential future researchers towards it (lines 414-419).

**Organization and Writing**

In the Introduction, Reviewer 2 asked for a more nuanced discussion of the meaning of race that more adequately reflects its complexity. We thank the reviewer for pointing out the shortcomings of this section, as we did not intend to imply that the entire scientific community had concluded that race is purely a social construct or that it has no association with biological factors. We have edited this text (lines 49-56) to acknowledge this point and to clarify that socially constructionist views of race still acknowledge that those categories are partly based in biological or descent-based characteristics. We have also added in several additional references and thank the reviewer for providing them.

Reviewer 3 asked us to introduce earlier in the paper that we would examine specific ancestry results on GATs as an influence on genetic essentialism. We added this information to the abstract and also to the Introduction (lines 97-100).

**Adding or Clarifying Statistics/Analyses**

Based on Reviewer 2’s helpful comments, we have revised our presentation of study population characteristics. We added a brief description of the sample’s characteristics to the main manuscript (lines 166-171) to provide context for the findings. We also edited the previously-confusing supplement table (now S2 Table) that presented these descriptive statistics. It now shows frequencies for categorical variables and means and standard deviations only for continuous variables.

We have clarified our hypotheses both about the role of genetic knowledge, and about the “confirmation” and “discovery” of new ancestry. We had only included what we expect the low genetic knowledge to experience as a result of seeing the test results, which is an increase in genetic essentialism. To complement the section, we added the following sentence in lines 130-133: "By contrast, we expect test-takers with high knowledge of genetics to be more likely to read the results as evidence that races and racial traits are not determined solely by genetics, and thereby develop less essentialist views after testing." We also specified that with regard to the specific ancestry test results, we expect a null main effect, but that high genetic literacy will lead to less essentialism while low genetic literacy will lead to more genetic essentialism (lines 134-146).

At Reviewer 3’s suggestion, we clarified the claim, regarding the additional survey item measuring genetic knowledge, that “models that included this item showed similar results…” (now lines 220-221) to specify that these are models that *we* ran with this item. We decided not to include the statistics for those additional models, both because PLOS One does not allow footnotes and because including this information would require adding a somewhat lengthy explanation of how we coded genetic knowledge using three items rather than two. Because the third genetic knowledge item (how much of the genes of a Black person are the same as those of a White person) has considerable theoretical overlap with our dependent variable, we feel that adding a lengthy discussion of this does not add value to the paper. If the Editor feels it is more appropriate, we can simply delete this sentence.

We have edited our ‘Analysis’ section (lines 246-268) to be clearer about the statistical techniques we are using and which models they draw from. We specified “Ordinary Least Squares” the first time we provided the abbreviation, on line 249. We also clarified that the statistics procedure used for the second hypothesis (i.e., testing the effects of specific test results on genetic essentialism controlling for genetic literacy) is OLS regression. We say this on line 337 in the updated manuscript.

We confirmed that all our models include control variables. In the text, we stated this on lines 266-268 but changed the wording a bit to make it clearer: “All models, both experimental and non-experimental, control for living in the South, interaction with non-Whites and political party preference, as well as gender, age, and education.”

We thank Reviewer 3 for noting the omission of the statistic being reported in several places on the former page 10. The numbers refer to average predicted probabilities, also known as marginal effects. We changed the text by adding “predicted probabilities” in the necessary parts in the results section. We also would like to state that the average predicted probabilities capture the effect sizes that are relevant to our analysis.

We added information to the notes of S1 Table and S10 Table to explain what variables are added to each of these nested regression models. All variables in the model are shown in the left-hand column, so we did not add a footnote with this information. We also added information to the manuscript to explain what Models 1 and 2 of S13 Table are (now lines 344-345).

**Figure Legends and Duplication of Information in Figures/Table**

The reviewers noted that there was some duplication of information in the figures, text, and the table in the manuscript. However, they differed on what should be done about it. Based on our best judgment, we decided to move the table (formerly Table 1) to the supplement (now S9 Table). We also added the sample sizes of each knowledge level in the control and treatment group to this table. We have kept the changes in essentialism scores in the text because the exact numbers may not be clear from the figures. We added a note to Figure 3 and clarified what the values refer to in the text. We also made slight changes by bringing some details from the notes of Figure 2 to the main text (lines 273-266).

**Minor Concerns**

We did not intend to set up the expectation that we would be examining the impact of GATs on multiple types of essentialism. We edited the wording in the Introduction (lines 56-58) to clarify that here we were distinguishing between genetic essentialism and cultural essentialism or other types of essentialism that we do not study. We have also removed the names of the three sub-factors that contribute to the full genetic essentialism scale (lines 195-198). We wanted to indicate that this is a complex concept that a second-order model helps to capture, but removed these titles to avoid setting up readers to expect that we would be analyzing the sub-factors individually.

Reviewer 2 asked for a citation for the GKAP study. The Co-Principal Investigator sent us the reference that we include at the bottom of this memo. However, because PLOS One does not allow citations to unpublished works, we decided to instead cite a publication by the Co-PIs that uses the GKAP and discusses the survey details (line 213, citation 50).

We have made all the minor editorial corrections pointed out by the reviewers, with the exception of referring to the Supplemental tables using the same pattern as for the main tables and figures (e.g. Table S1 instead of S1 Table). Here we are following the PLOS One formatting guidelines.

**Editors’ Comment**

We have provided permission from the copyright holder to reproduce S1 Fig and S2 Fig.

Once again, we would like to thank the Reviewers for reading the paper so carefully and for their very helpful comments. We very much hope that the revisions to the manuscript adequately address the issues that were raised.

References:

Liddell, Torrin M. & John K.Kruschke. 2018. Analyzing ordinal data with metric models: What could possibly go wrong? *Journal of Experimental Social Psychology* 79: 328-348.

Survey on Genomics Knowledge, Attitudes, and Policies (GKAP), by Jennifer Hochschild and Maya Sen. GKAP 1 in 2011; GKAP 2 in 2017. GKAP 1 was funded by Robert Wood Johnson Foundation, as part of a Investigator Award in Health Policy Research, 2010-2013 (with Maya Sen). Surveys were conducted by Knowledge Networks (now GfK).
